# Histone Deacetylase Inhibitors Improve the Replication of Oncolytic Herpes Simplex Virus in Breast Cancer Cells

**DOI:** 10.1371/journal.pone.0092919

**Published:** 2014-03-20

**Authors:** James J. Cody, James M. Markert, Douglas R. Hurst

**Affiliations:** 1 Department of Pathology, University of Alabama at Birmingham, Birmingham, Alabama, United States of America; 2 Department of Neurosurgery, University of Alabama at Birmingham, Birmingham, Alabama, United States of America; Geisel School of Medicine at Dartmouth, United States of America

## Abstract

New therapies are needed for metastatic breast cancer patients. Oncolytic herpes simplex virus (oHSV) is an exciting therapy being developed for use against aggressive tumors and established metastases. Although oHSV have been demonstrated safe in clinical trials, a lack of sufficient potency has slowed the clinical application of this approach. We utilized histone deacetylase (HDAC) inhibitors, which have been noted to impair the innate antiviral response and improve gene transcription from viral vectors, to enhance the replication of oHSV in breast cancer cells. A panel of chemically diverse HDAC inhibitors were tested at three different doses (<,  = , and >LD_50_) for their ability to modulate the replication of oHSV in breast cancer cells. Several of the tested HDAC inhibitors enhanced oHSV replication at low multiplicity of infection (MOI) following pre-treatment of the metastatic breast cancer cell line MDA-MB-231 and the oHSV-resistant cell line 4T1, but not in the normal breast epithelial cell line MCF10A. Inhibitors of class I HDACs, including pan-selective compounds, were more effective for increasing oHSV replication compared to inhibitors that selectively target class II HDACs. These studies demonstrate that select HDAC inhibitors increase oHSV replication in breast cancer cells and provides support for pre-clinical evaluation of this combination strategy.

## Introduction

The metastasis of breast cancer to distant organs remains the most challenging aspect for the clinical management of this disease. As the five-year survival rate for patients with distant metastases at the time of diagnosis is less than 25% [1], it is clear that new treatments are needed for metastatic breast cancer. Existing therapies are limited in their effectiveness and can cause undesired side effects. As examples, two recent studies have underscored the long term risks of heart disease posed to breast cancer patients treated with either trastuzumab or anthracycline chemotherapeutics [2] as well as radiotherapy [3]. In contrast, oncolytic viruses have been proposed as a therapy that potentially avoids these long term risks due to their ability to selectively replicate in and destroy tumor cells while sparing normal cells [4]. Among the many oncolytic viruses under investigation, oncolytic herpes simplex virus (oHSV) has several advantages and is one of the most well studied [5]. While much of the initial interest in oHSV focused on its use as a therapy for brain tumors, an increasing number of preclinical studies have demonstrated that oHSVs can be effective against a variety of tumor types, including breast cancer. The safety of this approach has been established for several different cancers. However, these clinical trials have also illustrated the need for greater antitumor efficacy. For this reason, there is growing interest in the combination of oHSV with other treatment modalities, such as radiotherapy or chemotherapy, in an effort to enhance viral efficacy [6], [7].

Many cancers, including breast cancer, exhibit aberrant histone deacetylase (HDAC) expression or activity. HDAC inhibitors have been found to exert multiple antitumor effects, paving the way for clinical trials of these agents in several cancers, including breast cancer [8]. Two inhibitors, vorinostat and romidepsin, have recently been approved by the U.S. Food and Drug Administration (FDA) for the treatment of cutaneous T-cell lymphomas. Of particular interest to the field of oncolytic virotherapy, it has been recognized that HDAC inhibitors can also suppress expression of interferon response genes [9]. oHSV is commonly generated through deletion of the diploid γ_1_34.5 gene, which attenuates the virus in non-cycling cells. The γ_1_34.5 gene encodes the primary neurovirulence factor [10], which enables the virus to overcome the host cell's protein kinase R-mediated block of late viral protein translation and also contributes to the ability of HSV to inhibit host cell interferon response. Consequently, it has been shown in a limited number of studies that HDAC inhibitors may have the ability to improve oHSV virotherapy [11]–[13].

In this report, we screened a panel of HDAC inhibitors comprising several different chemical classes for their potential to augment the replication of oHSV in breast cancer cells. Because many of these inhibitors have not been tested in the cell lines used in this study, we first determined LD_50_ values for each inhibitor. Viral replication was assessed by pre- and co-treatment with the HDAC inhibitors at concentrations greater than, less than, and near their LD_50_ and at both high and low multiplicity of infection (MOI). Select HDAC inhibitors improved oHSV replication in the cancer cells but not in normal cells. Because many of these HDAC inhibitors and oHSV constructs are being evaluated in clinical trials, this combination may be an effective strategy for the treatment of metastatic breast cancer.

## Materials and Methods

### Chemicals

HDAC inhibitors (**Table 1**) were purchased from the following: belinostat (Cat. No. S1085; CAS No. 414864-00-9, Selleck Chemicals, Houston, TX), entinostat (E-3866; CAS No. 209783-80-2) and panobinostat (P-3703; CAS No. 404950-80-7, LC Laboratories, Woburn, MA), and remaining compounds were from Sigma-Aldrich, St. Louis, MO: APHA (3-(4-aroyl-1H-2-pyrrolyl)-N-hydroxypropenamide) compound 8 (A2478; CAS 676599-90-9), MC1568 (M1824; CAS No. 852475-26-4), 1-naphthohydroxamic acid (SML0078; CAS 6953-61-3), SAHA (suberoylanilide hydroxamic acid, Vorinostat; SML0061; CAS 149647-78-9), sodium butyrate (B5887; CAS No. 156-54-7), trichostatin A (T-8552; CAS No. 58880-19-6), tubastatin A hydrochloride (SML0044; CAS 1310693-92-5), valproic acid (PHR1061; CAS 99-66-1). Valproic acid was supplied as a solution whereas all other inhibitors were supplied in powdered form. A concentrated stock solution of sodium butyrate was prepared in sterile water. Concentrated stock solutions of the remaining inhibitors were prepared in dimethyl sulfoxide (DMSO; Sigma-Aldrich).

**Table 1:** HDAC Inhibitors Used in This Study.

Inhibitor	Chemical type	Selectivity	Potency
APHA Compound 8 (APHA 8)	hydroxamic acid	Class I	μM
Belinostat (BEL), PDX101	hydroxamic acid	Pan	μM
Entinostat (ENT), MS-275	benzamide	Class I^1^	μM
MC1568	hydroxamic acid	Class II	nM
1-Naphtholhydroxamic Acid (1NHA)	hydroxamic acid	HDAC 8	μM
Panobinostat (PAN), LBH-589	hydroxamic acid	Pan	nM
Sodium Butyrate (NaB)	short chain fatty acid	Class I, IIa	mM
Suberoylanilide Hydroxamic Acid (SAHA), Vorinostat	hydroxamic acid	Pan	μM
Trichostatin A (TSA)	hydroxamic acid	Pan	nM
Tubastatin A (TBSA)	benzamide	HDAC 6	nM
Valproic Acid (VPA)	short chain fatty acid	Class I, IIa	mM

1 Also inhibits the Class IIa enzyme HDAC 9.

### Cells and Viruses

The human metastatic breast cancer cell line MDA-MB-231 was described previously [14]. MCF10A is an immortalized human mammary epithelial cell line described previously [15]. The 4T1 murine mammary carcinoma cell line and the Vero African Green Monkey kidney cell line were obtained from American Type Culture Collection (Manassas, VA). MDA-MB-231 and 4T1 cells were maintained in a 1∶1 (v/v) mixture of Dulbecco's modified Eagle medium and Ham's F12 (DMEM/F12; Life Technologies, Carlsbad, CA) supplemented with 2 mM L-glutamine (Life Technologies) and 5% v/v FBS (Life Technologies). MCF10A cells were maintained in DMEM/F12 supplemented with 5% FBS, 10 ng/ml human epidermal growth factor, 100 ng/ml cholera toxin, 10 μg/ml insulin, 500 ng/ml hydrocortisone (Sigma-Aldrich), 2 mM L-glutamine (Life Technologies), and 1X non-essential amino acids (Life Technologies). Vero cells were maintained in Eagle's minimum essential medium, alpha modification (α-MEM; Sigma-Aldrich) supplemented with 7% FBS, 2 mM L-glutamine, 100 U/ml penicillin, and 100 μg/ml streptomycin (Mediatech, Manassas, VA).

M002 is a genetically-engineered human herpes simplex virus (HSV) that is derived from the HSV-1 (F) clinical isolate. M002 lacks both copies of the γ_1_34.5 neurovirulence gene and expresses murine interleukin 12 (IL-12) under the early growth response-1 promoter, and has been described previously [16].

### Cell viability assays

MDA-MB-231, 4T1 and MCF10A were seeded in 96-well plates at 1000 cells per well. Working dilutions of the HDAC inhibitors were prepared in DMEM/F12, 5% FBS and each dilution was added to the cells in triplicate, with five dilutions tested per inhibitor. The cells were allowed to incubate for 3 days and viability was assessed by a 2 hour incubation with AlamarBlue reagent (Life Technologies), according to the manufacturer's instructions. Fluorescence was determined at 570/580 nm excitation/emission with a Hitachi F-7000 fluorescence spectrophotometer. Dose-response curves and the dose lethal to 50% of the cells (LD_50_) were calculated using SigmaPlot 10.0 software.

### Viral replication assays

MDA-MB-231, 4T1, and MCF10A cells were seeded in 24-well plates at 50,000 cells/well and treated with HDAC inhibitors either 6 hours prior to (pre-treatment) or immediately following (co-treatment) viral infection. Inhibitors were diluted as above and added to the media at low (<LD_50_), middle (near LD_50_) and high (>LD_50_) doses (**Table 2**). Cells were infected with M002 at a multiplicity of infection (MOI) of 0.1 and 10 plaque-forming units (PFU) per cell as previously described [17]. Briefly, cells were rinsed with PBS, infected with M002 diluted in DMEM/F12 with 1% FBS for 2 hours. At 48 hours post-infection, cells and media were harvested and subjected to three freeze/thaw cycles followed by sonication. This time point was chosen to assay replication at the maximum level. Vero cells were then infected with serial dilutions of each sample. After 48 hours, plaques were stained with 1% w/v crystal violet (Sigma-Aldrich) in 70% ethanol and quantified. The titers from the HDAC inhibitor-treated samples were normalized to infected, untreated cells and expressed as fold change +/- the standard error.

**Table 2:** Doses of HDAC Inhibitors Used in Viral Replication Experiments.

	LOW	MID	HIGH
**APHA 8**	1 μM	10 μM	50 μM
**BEL**	0.01 μM	0.25 μM	1 μM
**ENT**	0.1 μM	1 μM	5 μM
**MC1568**	10 μM	50 μM	100 μM
**NaB**	0.1 mM	1 mM	10 mM
**1-NHA**	10 μM	50 μM	100 μM
**PAN**	5 nm	10 nm	100 nm
**SAHA**	0.1 μM	1 μM	10 μM
**TBSA**	1 μM	50 μM	100 μM
**TSA**	0.1 μM	0.25 μM	1 μM
**VPA**	1 mM	10 mM	50 mM

## Results

### HDAC inhibitor LD_50_ values in breast cancer cells

**Figure 1:**
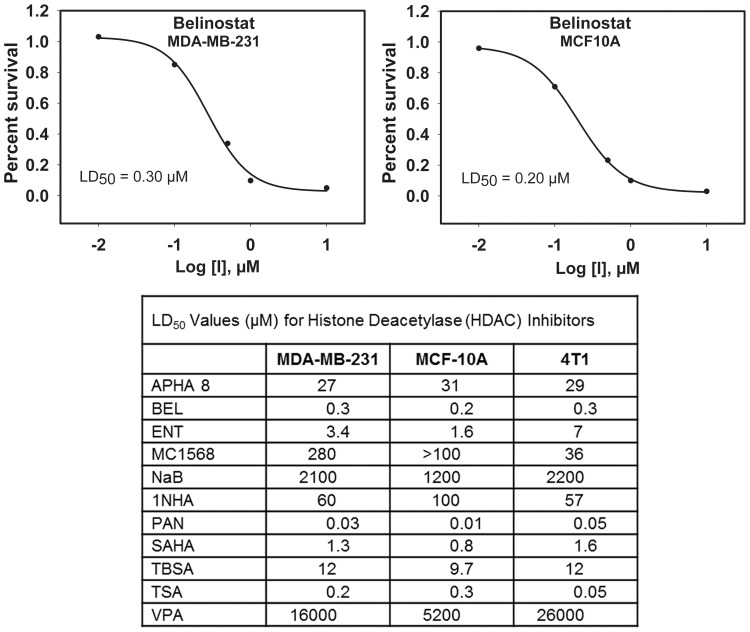
Approximate LD_50_ values determined for a panel of HDAC inhibitors in breast cancer cells. Proliferating breast cancer (MDA-MB-231), murine mammary carcinoma (4T1) and normal breast epithelial (MCF10A) cells were treated with a panel of histone deacetylase inhibitors at a range of concentrations, and cell viability was assessed after three days. Shown are representative dose-response curves for MDA-MB-231 and MCF10A cells treated with belinostat (upper panels) and a table of approximate LD_50_ values calculated from dose-response curves for the entire panel of inhibitors.

Prior to analyzing the effect of HDAC inhibitors on the replication of oHSV, it was first necessary to determine LD_50_ values since those have not been reported in the breast cancer cells used in this study for most of the inhibitors. We obtained a panel of inhibitors representing several different chemical classes, HDAC selectivities, and potencies (**Table 1**). An emphasis was placed on compounds currently under clinical investigation or in clinical use. LD_50_ values were calculated for each inhibitor in MDA-MB-231, 4T1 and MCF10A cell lines (**Figure 1**). Generally, the LD_50_ values were similar in all three cell lines and for most compounds were in the micromolar range. Three inhibitors, BEL, PAN, and TSA (all hydroxamic acids), yielded LD_50_ values in the submicromolar range. In contrast, the LD_50_ values for the two short chain fatty acids (NaB and VPA) were in the millimolar range.

### Pre-treatment of breast cancer cells with HDAC inhibitors enhances the replication of oHSV

**Figure 2:**
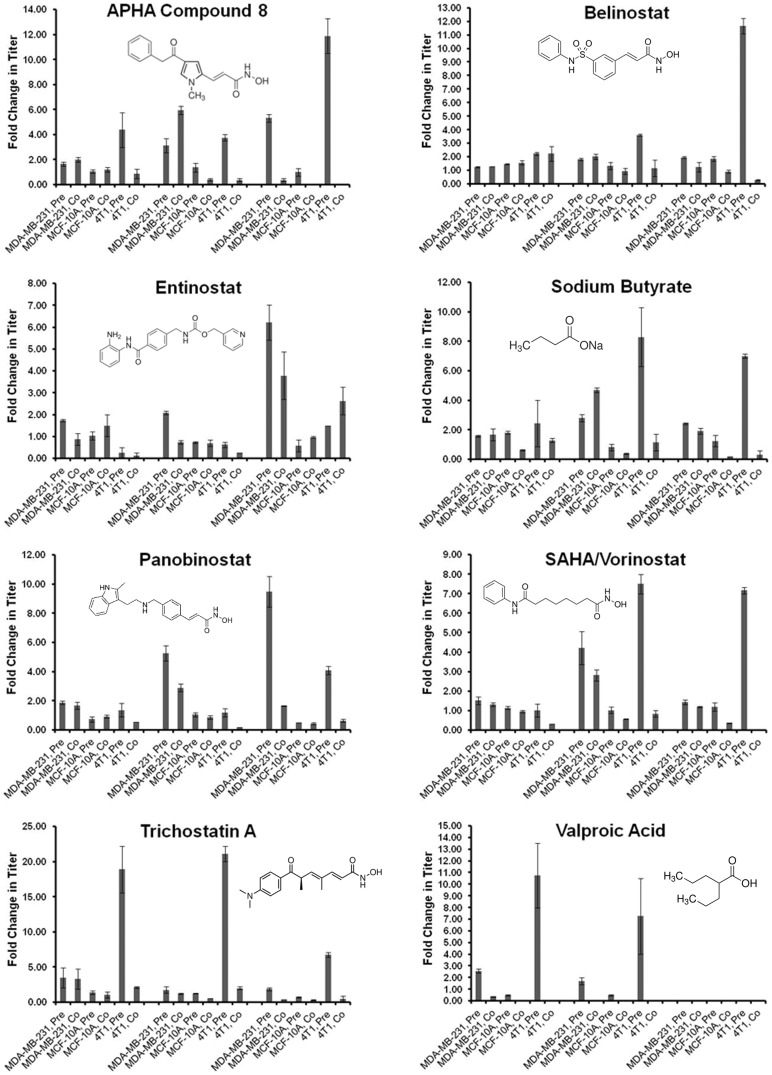
HDAC inhibitors enhance oHSV replication in breast cancer cells, but not in normal breast epithelial cells. MDA-MB-231 human breast cancer, MCF10A normal breast epithelial, and 4T1 murine mammary carcinoma cells were treated with the indicated HDAC inhibitors either 6 hours prior (Pre-) or immediately following (Co-) infection with M002 oHSV at 0.1 PFU/cell. Shown are fold changes in viral titer versus replication in untreated cells, at 48 hours post infection. From left to right, the three sets of bars within each graph indicate inhibitor concentrations below LD_50_, near LD_50_ and above LD_50_.

Having determined LD_50_ values for our panel of HDAC inhibitors, we then sought to examine how treatment of breast cancer cells with these inhibitors modulated the replication of the γ_1_34.5-deleted oHSV M002. The inhibitors were added to the cells at three different doses: a low dose (<LD50), middle dose (near the LD_50_) and a high dose (>LD50), as listed in **Table 2**. The same doses were used for all cell lines since the LD_50_ values were comparable. Two treatment schemes were utilized: six hours prior to viral infection (designated pre-treatment) and immediately following viral infection (co-treatment). After 48 hours, cells with media were harvested and the titers of plaque-forming units per ml were determined by standard plaque assay. Increases in replication were calculated as the fold change in titer of HDAC inhibitor-treated samples relative to infected, untreated cells (titers averaged 1.5×10^7^ and 1.4×10^5^ PFU/ml for MDA-MB-231 and 4T1, respectively). HDAC inhibitor treatment did not increase replication in cells infected at a high MOI (10 PFU/cell; data not shown). However, in cells infected at low MOI (0.1 PFU/cell), examination of fold changes revealed a number of trends. Most of the tested compounds increased replication at least 2 fold in the MDA-MB-231 cells, whereas replication in the normal breast cell line was not significantly affected (fold changes +/− standard error are given for all inhibitors in **Table S1**). In the MDA-MB-231 cells, some inhibitors enhanced replication as both a pre-treatment and a co-treatment (low dose: TSA; mid dose: APHA8, NaB, PAN, SAHA; high dose: ENT), but with the exception of APHA8 and NaB (mid dose) the highest magnitude of increase for each of these inhibitors was obtained by pre-treatment. Additionally, for some inhibitors (such as ENT, VPA, TBSA, 1NHA), replication was reduced by co-treatment at certain doses.A few compounds were particularly effective at increasing replication in the MDA-MB-231 cells, with fold changes greater than 5 for APHA8 (mid, high doses), ENT (high dose), and PAN (mid, high doses; **Figure 2**). The greatest increase (approximately 1 log) in viral replication was obtained by pre-treatment with PAN at the highest dose.

### HDAC inhibitors enhance the replication of oHSV in an HSV-resistant cancer cell line

In a previous study, we have shown that while other mouse carcinoma cell lines are similarly permissive for oHSV as human cancer cells, the aggressive murine mammary carcinoma 4T1 cell line is relatively resistant to γ_1_34.5-deleted oHSVs [18], a result that has also been shown by others [19]. Having observed that HDAC inhibitor treatment enhanced oHSV replication in tumor cells but not normal cells, we postulated that HDAC inhibitor treatment might make an oHSV-resistant line more susceptible to viral replication. We selected 4T1 as a representative oHSV-resistant carcinoma cell line. With the exception of MC1568, all of the inhibitors increased oHSV replication in the 4T1 cells, particularly with pre-treatment at the mid and high doses (**Figure 2** and **Table S1**). In fact, the increases were more pronounced than those observed in the MDA-MB-231 cells. As with the MDA-MB-231 cells, pre-treatment yielded the highest magnitude in fold increase for most of the effective compounds (with the exception of ENT) whereas co-treatment frequently yielded reduced replication at the mid and high doses in the 4T1 cells. Many of the HDAC inhibitors tested yielded increases in replication greater than 5 fold (low dose: TSA, VPA; mid dose: NaB, SAHA, TSA, VPA; high dose: APHA8, BEL, NaB, SAHA, TSA) and several yielded increases greater than 10 fold (APHA 8, BEL, TSA, VPA), with the highest increase (>20 fold) obtained by TSA at the mid dose. Overall, these data indicate that select HDAC inhibitor treatment can render an oHSV-resistant cell line more susceptible to viral replication.

## Discussion

There remains an urgent need for more effective therapies for metastatic breast cancer. Here, we have investigated the potential utility of combining two developing therapeutics: oHSV and HDAC inhibitors. We have determined LD_50_ values for a panel of inhibitors that includes compounds belonging to several different chemical classes to directly compare cell death in metastatic breast cancer versus normal mammary epithelial cells. In general, the LD_50_ values were in the same range for all of the cell lines tested. Several of the inhibitors have not been previously tested in breast cancer cells, and a comprehensive comparison of multiple compounds in these cells has not previously been reported.

In the viral replication experiments, we characterized the ability of HDAC inhibitors to enhance the replication of γ_1_34.5-deleted oHSV in breast cancer cells. Although oHSVs with a variety of mutations have been described, those based on mutation of the γ_1_34.5 gene have advanced the furthest in clinical testing. We selected the γ_1_34.5-deleted oHSV M002 due to its enhanced antitumor potency versus non-cytokine oHSVs such as G207 [20], [21], which has been evaluated in Phase I [22] and Phase Ib [23] clinical trials. M002 expresses murine IL-12 to promote an adaptive antitumor immune response and carries a wild type ribonucleotide reductase gene, enabling more efficient replication in cancer cells than G207 [21]. All of the inhibitors in this study mediated at least modest (>2 fold) increases in viral replication in the cancer cell lines, although for some compounds this increase may have been within the range of titration error. The most effective compounds (fold changes >5) were broad-spectrum inhibitors APHA8, BEL, ENT, NaB, PAN, SAHA, TSA, and VPA. In contrast, the least effective inhibitors included the isoform specific compounds MC1568 (Class II HDACs), 1NHA (HDAC 8), and TBSA (HDAC 6).Although not conclusive, these data suggest that broad-spectrum HDAC inhibitors, particularly those that inhibit Class I HDACs, are likely to be more useful than class-specific compounds at enhancing oHSV replication. However, mechanistic studies will be needed to determine which individual HDAC(s) might be the most critical for inhibition.

The most detailed studies of HDAC inhibitors used in combination with oHSV have been in the context of glioma [12], [13], [24], colon cancer [12] and squamous cell carcinoma [11]. The potential for use against breast cancer has not been extensively explored, although Liu *et al.* reported an additive cytotoxic effect of TSA in combination with oHSV in the MCF-7 cell line that was not due to increased viral replication [12]. In those studies, detailed analyses have only been conducted with TSA [11]–[13] and VPA [13], [24]. The replication of other oncolytic viruses including vesicular stomatitis virus [25], vaccinia virus [25], [26] and others [27] have also been shown to be enhanced by some HDAC inhibitors.

In this study, we provide further evidence that the timing of administration is important, as pre-treatment was found to yield higher increases in viral replication than co-treatment. Similarly, Otsuki *et al.* report that pre-treatment with VPA enhances viral replication, but co-treatment does not [13]. Katsura *et al.* demonstrated that co-treatment with TSA enhanced replication at 24 hours post-infection but gave no benefit at 12 or 36 hours, and did not examine pre-treatment [11]. The effectiveness of particular treatment schedules may also be cell line specific. In our study, treatment schedule appeared to have little influence on enhancement of viral replication in the TSA-treated MDA-MB-231 cells, but greatly influenced replication in the oHSV-resistant 4T1 cells.

For some HDAC inhibitors, viral replication was reduced to the extent that no plaques were detected at the dilutions assayed (resulting in “not determined”, **Table S1**). This may have been the result of cell death limiting viral replication, since this was most often seen with co-treatment at the mid and high doses. Nonetheless, increased replication was observed for some inhibitors even at concentrations above the LD_50_ (particularly for pre-treatment). This is presumably because a dose sufficient to kill cells over a 72 hour treatment (as in the determination of the LD_50_ values) is not lethal within the time frame of the viral replication experiments (48 h). Although co-treatment lead to decreased replication in some cases, it is possible that synergistic cell death from combined oHSV/HDAC inhibitor treatment would result in enhanced antitumor effect *in vivo*. Whether pre-treatment or co-treatment is more advantageous therapeutically will have to be determined in an *in vivo* system, and may depend on the inhibitor used.

Work by other investigators has identified several mechanisms by which HDAC inhibitors enhance the antitumor efficacy of oHSV, both at the cellular level and at the level of the tumor microenvironment (reviewed by Nguyen *et al.* [27]). In a glioma model, VPA was shown to enhance oHSV gene expression and inhibit the expression of interferon-responsive genes, enabling increased viral replication [13]. Studies focused on TSA in combination with oHSV have shown that a reduction in cyclin D1 levels [12] and enhancement of nuclear factor kappaB (NFκB) activation via p65 acetylation [11] are also contributing factors. Additional studies are needed to identify other potential mechanisms, such as changes in the efficiency of infection or modification of viral genome-associated histones.

Two HDAC inhibitors (TSA and VPA) have been tested *in vivo* with oHSV [12], [13]. In murine models of both glioma and colon cancer, the combination of oHSV with TSA and VPA, respectively, has been shown to enhance antitumor effect over oHSV alone. Because HDAC inhibitors exert a variety of antitumor effects independent of their effect on oncolytic viral replication, and because the combination of these two treatment strategies can yield synergistic effects, this could allow for the administration of lower inhibitor doses, thereby avoiding the toxicities of HDAC inhibitors as a monotherapy.

One potential concern is that HDAC inhibitors might mitigate the cancer-selective replication of an oHSV and enable replication in otherwise non-permissive normal cells. However, our results show that HDAC inhibitor treatment did not enhance replication of M002 in the MCF10A normal mammary epithelial cells. A lack of increased oHSV replication has also been noted in primary prostate epithelial cells and quiescent endothelial cells [12] as well as normal human astrocytes [13]. It is unclear why HDAC inhibitors selectively increase oHSV replication in cancer cells but not normal cells, but tumor cell reliance on aberrant HDAC activity or the full complement of antiviral pathways active in normal cells are two possibilities. An additional safety concern is that HDAC inhibitor treatment might enhance the replication of wild-type HSV. Liu *et al.* showed that the combination of TSA and wild-type HSV yielded no effect on viral replication in quiescent cells or normal primary prostate epithelial cells [12]. Additionally, mice given intracranial wild-type HSV showed no enhancement of encephalitis by VPA administration [24]. In additional experiments, we have similarly observed that wild-type HSV replication was not enhanced by HDAC inhibitors in MCF10A cells, although it was increased in the MDA-MB-231 and 4T1 cell lines (data not shown). Overall, these results indicate that the use of HDAC inhibitors is unlikely to compromise the excellent safety record of oHSV.

In conclusion, we have shown that HDAC inhibitors can be used to increase the replication of an oHSV in breast cancer cells without increasing replication in normal breast epithelial cells, and we have shown that an oHSV-resistant tumor cell line can be made more susceptible to oHSV replication. The magnitude of replication increase is dependent upon cell line, treatment schedule, viral MOI, and inhibitor dose. We did not observe that a particular chemical class of inhibitor was uniformly more effective than others, nor did we observe that a single inhibitor was the most effective in both cancer cell lines. Rather, our data suggest that the selectivity profile of the inhibitor is the most important determinant in how well oHSV replication is enhanced. Our data indicate that broad spectrum inhibitors or those that inhibit Class I HDACs in particular are more effective for increasing viral replication than selective inhibitors targeting class II HDACs (**Table 3**). Of the inhibitors we tested, 8 increased oHSV replication >5 fold. Of these, 5 are currently being evaluated in clinical trials for breast cancer (BEL, PAN, SAHA, ENT, VPA). This is encouraging for the purposes of clinical relevance, since it provides several potential combinations to pursue.

**Table 3:** Summary of Increased oHSV Replication in Cancer Cell Lines Pre-treated with HDAC Inhibitors.

Inhibitor Selectivity	Compound	Clinical Status^1^	Replication Increase				
			MDA-MB-231		4T1		
			MID	HIGH		MID	HIGH
Pan	BEL	Phase I	−	−		+	+++
	PAN	Phase II	++	++		−	+
	SAHA^2^	Phase II	+	−		++	++
	TSA	Preclinical	−	−		+++	++
Class I	APHA 8	Preclinical	++	++		+	+++
	ENT^3^	Phase II	+	++		−	+
Class I and IIa	VPA	Phase II	−	−		++	−
	NaB	Preclinical	+	+		++	++
Class II	MC1568	Preclinical	−	−		−	−
HDAC 6 (*Class IIb*)	TBSA	Preclinical	+	−		−	+
HDAC 8 (*Class I*)	1-NHA	Preclinical	−	−		−	+

No increase (-) or increases in replication of >2 fold (+), >5 fold (++) and >10 fold (+++) are shown.

1 For breast cancer.

2 Clinically approved for the treatment of cutaneous T cell lymphoma.

3 Also inhibits the Class IIa enzyme HDAC 9.

## Supporting Information

Table S1 Fold changes in viral titer (+/− standard error) in cell lines pre-treated or co-treated with the indicated HDAC inhibitors, normalized to titer from untreated cells.
(DOCX)
