# Supplementary material for: Histone Deacetylase Inhibitors Improve the Replication of Oncolytic Herpes Simplex Virus in Breast Cancer Cells
Source: PLoS One. 2014 Mar 20;9(3):e92919. doi: 10.1371/journal.pone.0092919 (PMC3961437; doi:10.1371/journal.pone.0092919)
Supplement: Table S1 — Fold changes in viral titer (+/− standard error) in cell lines pre-treated or co-treated with the indicated HDAC inhibitors, normalized to titer from untreated cells. (DOCX) [file pone.0092919.s001.docx]

TABLE S1.

| **Table S1.** Fold changes in viral titer (+/- standard error) in cell lines pre-treated or co-treated with the indicated HDAC inhibitors, | | | | | | | | | | | | | | | | | | |
| --- | --- | --- | --- | --- | --- | --- | --- | --- | --- | --- | --- | --- | --- | --- | --- | --- | --- | --- |
|  | normalized to titer from untreated cells | | | | | |  |  |  |  |  |  |  |  |  |  |  |  |
|  |  |  |  |  |  |  |  |  |  |  |  |  |  |  |  |  |  |  |
|  | Low Dose (< LD_50_) | | | | | | Middle Dose (Near LD_50_) | | | | | | High Dose (> LD_50_) | | | | | |
|  | **MDA-MB-231** | | **MCF10A** | | **4T1** | | **MDA-MB-231** | | **MCF10A** | | **4T1** | | **MDA-MB-231** | | **MCF10A** | | **4T1** | |
|  | Pre | Co | Pre | Co | Pre | Co | Pre | Co | Pre | Co | Pre | Co | Pre | Co | Pre | Co | Pre | Co |
| **APHA8** | 1.66 | 2.00 | 1.05 | 1.22 | 4.38 | 0.88 | 3.13 | 5.94 | 1.38 | 0.41 | 3.75 | 0.38 | 5.31 | 0.38 | 1.00 | 0.05 | 11.88 | ND |
| +/- | 0.16 | 0.19 | 0.14 | 0.19 | 1.38 | 0.38 | 0.56 | 0.31 | 0.35 | 0.08 | 0.25 | 0.13 | 0.31 | 0.13 | 0.30 | 0.01 | 1.38 | 0.00 |
| **BEL** | 1.25 | 1.30 | 1.44 | 1.56 | 2.22 | 2.22 | 1.80 | 2.00 | 1.33 | 0.93 | 3.61 | 1.17 | 1.95 | 1.25 | 1.84 | 0.91 | 11.67 | 0.28 |
| +/- | 0.05 | 0.00 | 0.05 | 0.16 | 0.11 | 0.56 | 0.10 | 0.20 | 0.26 | 0.23 | 0.06 | 0.61 | 0.05 | 0.35 | 0.16 | 0.12 | 0.56 | 0.06 |
| **ENT** | 1.73 | 0.86 | 1.04 | 1.50 | 0.25 | 0.13 | 2.08 | 0.73 | 0.73 | 0.69 | 0.63 | 0.25 | 6.22 | 3.78 | 0.58 | 0.96 | 1.50 | 2.63 |
| +/- | 0.05 | 0.27 | 0.19 | 0.50 | 0.25 | 0.13 | 0.08 | 0.08 | 0.04 | 0.15 | 0.13 | 0.00 | 0.81 | 1.08 | 0.27 | 0.04 | 0.00 | 0.63 |
| **MC1568** | 4.85 | 1.82 | 1.35 | 0.94 | 0.17 | 0.08 | 1.67 | 1.09 | 1.41 | 0.90 | 0.33 | ND | 1.85 | 0.58 | 1.02 | 0.63 | 0.25 | ND |
| +/- | 0.61 | 0.12 | 0.06 | 0.20 | 0.00 | 0.08 | 0.03 | 0.18 | 0.12 | 0.24 | 0.17 | 0.00 | 0.21 | 0.09 | 0.16 | 0.00 | 0.08 | 0.00 |
| **NaB** | 1.58 | 1.67 | 1.81 | 0.62 | 2.43 | 1.29 | 2.79 | 4.70 | 0.81 | 0.37 | 8.29 | 1.14 | 2.42 | 1.91 | 1.24 | 0.20 | 7.00 | 0.29 |
| +/- | 0.06 | 0.39 | 0.10 | 0.05 | 1.57 | 0.14 | 0.24 | 0.15 | 0.24 | 0.04 | 2.00 | 0.57 | 0.06 | 0.21 | 0.38 | 0.00 | 0.14 | 0.29 |
| **1NHA** | 1.28 | 1.02 | 1.33 | 1.29 | 0.67 | 1.17 | 1.16 | 0.26 | 1.00 | 0.58 | 0.67 | 0.50 | 1.24 | 0.12 | 1.54 | 0.75 | 4.33 | ND |
| +/- | 0.20 | 0.02 | 0.25 | 0.21 | 0.33 | 0.50 | 0.16 | 0.02 | 0.33 | 0.01 | 0.00 | 0.17 | 0.12 | 0.04 | 0.04 | 0.08 | 1.00 | 0.00 |
| **PAN** | 1.87 | 1.66 | 0.73 | 0.92 | 1.36 | 0.55 | 5.26 | 2.89 | 1.05 | 0.86 | 1.18 | 0.18 | 9.47 | 1.66 | 0.49 | 0.43 | 4.09 | 0.64 |
| +/- | 0.13 | 0.24 | 0.19 | 0.11 | 0.45 | 0.00 | 0.53 | 0.26 | 0.14 | 0.11 | 0.27 | 0.00 | 1.05 | 0.03 | 0.00 | 0.05 | 0.27 | 0.09 |
| **SAHA** | 1.52 | 1.31 | 1.15 | 0.95 | 1.00 | 0.33 | 4.23 | 2.82 | 1.02 | 0.57 | 7.50 | 0.83 | 1.44 | 1.20 | 1.20 | 0.38 | 7.17 | ND |
| +/- | 0.20 | 0.10 | 0.07 | 0.07 | 0.33 | 0.00 | 0.85 | 0.28 | 0.16 | 0.02 | 0.50 | 0.17 | 0.11 | 0.04 | 0.21 | 0.02 | 0.17 | 0.00 |
| **TBSA** | 1.50 | 2.07 | 1.14 | 1.23 | 1.25 | 1.00 | 2.11 | ND | 0.73 | 0.01 | 1.00 | ND | 1.61 | ND | 0.77 | ND | 2.25 | ND |
| +/- | 0.07 | 0.07 | 0.26 | 0.18 | 0.25 | 0.00 | 0.04 | 0.00 | 0.01 | 0.00 | 0.00 | 0.00 | 0.32 | 0.00 | 0.09 | 0.00 | 0.25 | 0.00 |
| **TSA** | 3.52 | 3.33 | 1.42 | 1.08 | 18.89 | 2.11 | 1.76 | 1.24 | 1.26 | 0.58 | 21.11 | 2.00 | 1.86 | 0.40 | 0.75 | 0.32 | 6.78 | 0.44 |
| +/- | 1.43 | 1.43 | 0.25 | 0.42 | 3.33 | 0.11 | 0.43 | 0.10 | 0.03 | 0.01 | 1.11 | 0.22 | 0.24 | 0.01 | 0.08 | 0.05 | 0.33 | 0.44 |
| **VPA** | 2.56 | 0.36 | 0.48 | 0.08 | 10.75 | ND | 1.69 | ND | 0.48 | ND | 7.25 | ND | ND | ND | ND | ND | ND | ND |
| +/- | 0.19 | 0.02 | 0.04 | 0.00 | 2.75 | 0.00 | 0.31 | 0.00 | 0.04 | 0.00 | 3.25 | 0.00 | 0.00 | 0.00 | 0.00 | 0.00 | 0.00 | 0.00 |
|  | ND = not determined | | |  |  |  |  |  |  |  |  |  |  |  |  |  |  |  |
